# Cervical cancer screening using HPV tests on self-samples: attitudes and preferences of women participating in the VALHUDES study

**DOI:** 10.1186/s13690-021-00667-4

**Published:** 2021-08-30

**Authors:** Hélène De Pauw, Gilbert Donders, Steven Weyers, Philippe De Sutter, Jean Doyen, Wiebren A. A. Tjalma, Davy Vanden Broeck, Eliana Peeters, Severien Van Keer, Alex Vorsters, Marc Arbyn

**Affiliations:** 1 Unit of Cancer Epidemiology, Belgian Cancer Centre, Sciensano, J. Wytsmanstreet 14, B1050 Brussels, Belgium; 2 Department of Obstetrics and Gynaecology of the General Regional Hospital Heilig Hart (RZ Tienen), Tienen, Belgium; 3 Femicare vzw, Clinical Research for Women, Tienen, Belgium; 4 Department of Obstetrics and Gynaecology, Antwerp University Hospital (UZA), Antwerp, Belgium; 5 Department of Obstetrics and Gynaecology, Ghent University Hospital (UZ Ghent), Ghent, Belgium; 6 Department of Human Structure and Repair, Faculty of Medicine and Health Sciences, University of Ghent, Ghent, Belgium; 7 Department of Gynaecology & Oncology, Universitair Ziekenhuis Brussel (UZ Brussel) - Vrije Universiteit Brussel (VUB), Brussels, Belgium; 8 Department Gynaecology-Obstetrics, Liège University Hospital (CHU Liège), Liège, Belgium; 9 Multidisciplinary Breast Clinic, Unit Gynaecologic Oncology, Department of Obstetrics and Gynaecology, Antwerp University Hospital (UZA), Edegem, Belgium; 10 Molecular Imaging, Pathology, Radiotherapy, Oncology (MIPRO), Faculty of Medicine and Health Sciences, University of Antwerp, Antwerp, Belgium; 11 Laboratory of Molecular Pathology, AML Sonic Healthcare, Antwerp, Belgium; 12 National Reference Centre for HPV, Brussels, Belgium; 13 AMBIOR, Laboratory for Cell Biology & Histology, University of Antwerp, Antwerp, Belgium; 14 International Centre for Reproductive Health, Ghent University, Ghent, Belgium; 15 Unit of Cancer Epidemiology, Belgian Cancer Centre, Scientific Institute of Public Health, Brussels, Belgium; 16 Centre for the Evaluation of Vaccination (CEV), Vaccine & Infectious Disease Institute (VAXINFECTIO), Faculty of Medicine and Health Sciences, University of Antwerp, Wilrijk, Antwerp, Belgium

**Keywords:** Cervical cancer, Screening, Attitudes, Preferences, Human papillomavirus, HPV, Self-sampling, Urine, VALHUDES

## Abstract

**Background:**

Interventions to reach women who do not participate regularly in screening may reduce the risk of cervical cancer. Self-collection of a vaginal specimen has been shown to increase participation. The relative clinical accuracy of human papillomavirus (HPV) testing on first-void urine (with Colli-Pee) and on vaginal self-samples versus on cervical clinician-collected samples is being investigated in the VALHUDES trial. The current study assesses attitudes and experiences regarding self-sampling among women enrolled in VALHUDES.

**Methods:**

Questionnaires from 515 women (age 25–64 years [*N* = 498]; < 25 [*N* = 10], age ≥ 65 [*N* = 3], enrolled between December 2017 - January 2020) referred to colposcopy because of previous cervical abnormalities and enrolled in VALHUDES (NCT03064087) were analysed.

**Results:**

Of the 515 participants, nearly all women confirmed that self-sampling may help in reaching under-screened women (93%). Nevertheless, 44% of the participants stated before starting collection that a clinician-collected sample is more effective than a self-collected sample. After self-sampling, the large majority of women (> 95%) declared that instructions for self-collection were clear, that collection was easy, and that they were confident about having performed the procedure correctly, for both urine and vaginal collection. However, a proportion of women found self-sampling unpleasant (9.5% [49/515] for urine collection; 18.6% [96/515] and 15.5% [80/515] for vaginal sampling with cotton swabs or plastic brushes, respectively). For their next screening round, 57% would prefer self-sampling whereas 41% opted for collection by a clinician. Among women preferring self-sampling, 53% would choose for urine collection, 38% for vaginal self-collection and 9% had no preference. Age did not modify preferences.

**Conclusion:**

We conclude that both urine and vaginal self-sampling are well accepted by women, with a preference for urine sampling. Although the large majority of women are confident in their ability to perform self-sampling, four to five over ten women preferred specimen collection by a clinician.

**Trial registration:**

The study VALHUDES was registered in ClinicalTrials.gov (identifier: NCT03064087).

**Supplementary Information:**

The online version contains supplementary material available at 10.1186/s13690-021-00667-4.

## Background

The success of a cervical cancer (CC) screening programme strongly depends on the participation of the target population. Women who are under-screened or never-screened are at higher risk for CC than those who participate regularly [1]. Socio-economically disadvantaged groups and women older than 45 years often are insufficiently screened [2]. Endeavours to increase screening-participation in hard-to-reach women may reduce this risk substantially. Several barriers have been identified such as the lack of a gynaecologist or family physician, time constraints and low access to health services, physical discomfort, lack of knowledge and confidence in the benefits of CC screening, and low perceived risk of CC [3–6].

Randomised population-based controlled trials have demonstrated that HPV-based CC screening offers higher protection against incident cervical precancer and cancer compared to cytology-based screening [7, 8]. An additional advantage of screening using HPV assays is that the test can be performed on a self-sample collected by the woman herself. Recent systematic reviews have shown that HPV DNA tests on self-samples compared to on clinician-taken cervical samples have similar accuracy to detect underlying cervical precancer, under the condition that validated PCR-based HPV assays are used [9, 10]. Moreover, randomised participation trials have shown that the offer of a self-sampling kit generates higher response rates than conventional invitations to have a screening specimen taken by a clinician [11]. However, the magnitude of the response is very heterogeneous among studies, which suggests that the impact of strategies including self-sampling may depend on local conditions [11]. HPV testing can also be performed on urine, in particular on first-void (FV) urine, which is more accurate than testing on a random or midstream sample [12]. Collection of urine has been indicated as less embarrassing than taking a cervico-vaginal self-sample [13].

The clinical accuracy of HPV testing on urine and on vaginal samples is being investigated in the VALHUDES trial (VALidation of HUman papillomavirus assays and collection DEvices for Self-samples and urine samples) (NCT03064087) [14]. The current study assesses attitudes and experiences about collection of a specimen for CC screening taken by woman herself or taken by a clinician among women enrolled in VALHUDES.

## Methods

### Study population

The VALHUDES study design has been described previously [14]. In brief, five Belgian colposcopy clinics (University Hospital of Antwerp (UZA), Brussels (UZ Brussels), Ghent (UZ Ghent), Liège (CHU de Liège), and the General Regional Hospital Heilig Hart Tienen (RZ Tienen)) had to enrol at least 500 consenting eligible women between the age of 25 and 64, who were referred for colposcopy because of previous cervical abnormalities (Atypical Squamous Cell where a High-grade squamous lesion cannot be excluded [ASC-H] or worse squamous cervical abnormalities, Atypical Glandular Cells [AGC], repeated low-grade abnormalities or HPV+ Atypical Squamous cells of Undetermined Significance [ASC-US]). Hysterectomised women, women with known pregnancy, non-consenting women and women that were not able to understand and to sign the informed consent were excluded.

A study package, sent to the women’s home address, contained an information brochure, an informed consent form and instructions on how to collect urine samples with the Colli-Pee® (Novosanis, Wijnegem, Belgium).

Urine was collected at home before the consultation at the colposcopy centre. Upon arrival at the colposcopy clinic, two dry vaginal self-samples were collected: first taken with the Multi-Collect swab (Abbott GmbH & Co. KG, Wiesbaden, Germany) and the second with a plastic brush. In about one-half of the study population, the plastic vaginal brush was the Evalyn-Brush (Rovers® Medical Devices B.V., Oss, The Netherlands) and in the other half the Qvintip (Aprovix AB, Uppsala, Sweden). At the start of the colposcopy examination, a cervical cell specimen was collected by a gynaecologist with a Cervex-Brush (Rovers Medical Devices B.V., Oss, the Netherlands).

### VALHUDES questionnaire

Study participants completed a written questionnaire on paper at the colposcopy clinic after having completed the vaginal self-samplings. It was available in three languages (Dutch, French and English) The first four questions investigated knowledge about HPV, HPV vaccination status and attitudes regarding HPV testing on self-samples whereas the subsequent eight questions concerned experiences and intentions regarding possible future use of self-samples. Reading and answering all the questions took about 10-15 min to be completed by the woman herself. The English version of the full questionnaire is included in the Supplementary appendix (see Appendix I Questionnaire). After completing the questionnaire, women were invited by the gynaecologist who then collected a cervical cell specimen and performed the colposcopy.

### Statistical methods

The paper forms containing the answers to the questionnaire were encoded manually into an Excel database. A full check was done after data entry by the encoder. In addition, an independent check of a 25% random selection of the answer forms was performed by a VALHUDES researcher to pick-up possible data entry errors.

Frequency distributions, showing also the number of missing values, were tabulated for each question. Associations between categorical variables were assessed by Pearson’s chi-square test, or Fisher’s Exact test when the expected count in at least one cell was lower than five. A test for symmetry was used to assess the similarity of answers regarding each of the self-sampling methods [15]. As the proportion of missing values was small (< 5%), it was decided to include the missing data in the descriptive statistics without data imputation [16]. Statistical analyses were conducted with STATA/SE 16.0 (STATAcorp, College Station, Texas). Statistical significance was accepted when *p*-values < 0.05.

## Results

### Population characteristics

Between the 31st of December 2017 and the 2nd of January 2020, 523 women were enrolled in the VALHUDES trial. The distribution of the number of enrolled patients per colposcopy centre was based on clinical information provided by the responsible gynaecologists. In total, questionnaires from 515 women were obtained and analysed. Four hundred ninety-eight belonged to the eligible VALHUDES target age group (age 25 – 64 years), whereas ten and three women were younger than 25 years or older than 64, respectively. The distribution of the number of enrolled patients per colposcopy centre and characteristics of the respondents enrolled in the study are described in Table 1.

**Table 1:** Characteristics of the respondents enrolled in the study (*N* = 515)

Characteristics	Number	(%)
Age (years) (*N* = 511*)		
5-years age groups		
15-19	1	(0.2)
20-24	9	(1.8)
25-29	96	(18.8)
30-34	79	(15.5)
35-39	60	(11.7)
40-44	72	(14.1)
45-49	71	(13.9)
50-54	56	(11.0)
55-59	41	(8.0)
60-64	23	(4.5)
65-69	2	(0.4)
70-74	1	(0.2)
Participants enrolled per colposcopy centre		
UZA	21	(4.1)
UZ Brussels	53	(10.3)
UZ Ghent	228	(44.3)
CHU Liège	42	(8.2)
RZ Tienen	171	(33.2)
Number of enrolments by calendar year (*N* = 512^†^)		
2018	257	(50.2)
2019	254	(49.6)
2020	1	(0.2)
Self-reported HPV vaccination status		
vaccinated	106	(65.2)
unvaccinated	336	(20.6)
unknown	65	(12.6)
missing value	8	(1.6)
Plastic vaginal brush devices		
Evalyn	238	(46.2)
Qvintip	274	(53.2)
missing value	3	(0.6)

*4 missing values

†3 missing values

*Abbreviations*: *UZA* Antwerp University Hospital, *UZ Brussels* Brussels University Hospital, *UZ Ghent* Ghent University Hospital, *CHU de Liège* Liège University Hospital, *RZ Tienen* General Regional Hospital Heilig Hart Tienen

### Questions on general knowledge about HPV, HPV vaccination status and opinions on HPV testing on self-samples

The responses to questions 1 to 4 are summarised in Tables 2, 3 and 4.

**Table 2:** General knowledge about HPV and HPV vaccination status, *N =* 515 [n (%)]

No.	Question	No	Yes	No opinion	Missing value
**1**	*Did you know – before you accepted to participate in the VALHUDES study – that cervical cancer is caused by the human papillomavirus (=HPV), a virus that is sexually transmitted?*	135 (26.2)	364 (70.7)	1 (0.2)	15 (2.9)
**12**	*Are you vaccinated against HPV?*	336 (65.2)	106 (20.6)	65 (12.6)	8 (1.6)

**Table 3:** Opinions about self-sampling, *N =* 515 [n (%)]

No.	Question	No	Yes	No opinion	Missing value
**2**	*Do you find self-sampling a good solution to reach more women who do not go to a general practitioner or gynaecologist for a Pap smear?*	9 (1.8)	481 (93.4)	13 (2.5)	12 (2.3)
**3**	*We would like to know your opinion about the following statements (assuming that the self-sample and the clinician-taken sample have similar accuracy)*				
**3a**	I find that a sample taken by a doctor is better than a self-sample	139 (26.9)	227 (44.1)	128 (24.9)	21 (4.1)
**3b**	I think that most women will choose a self-sample instead of going to a doctor	115 (22.3)	297 (57.7)	86 (16.7)	17 (3.3)
**3c**	Self-sampling is good for women who don’t have taken a Pap smear	15 (2.9)	462 (89.7)	22 (4.3)	16 (3.1)

**Table 4:** Expectation about women’s preferred method of self-sampling, *N =* 515 [n (%)]

No.	4
**Question**	*I think that most women prefer the following method of self-sampling (assuming that the self-sample and the clinician-taken sample have similar accuracy)*
Urine sample	363 (70.5)
Vaginal sample	33 (6.4)
Both urine and vaginal sample	25 (4.9)
No preference	46 (8.9)
No opinion	35 (6.8)
Missing answer	13 (2.5)

When participants were asked whether they knew before their participation in VALHUDES that HPV causes CC, 70.7% (364/515) answered they were aware of the association whereas 26.2% (135/515) were unaware. Sixty-five percent (336/515) reported that they were not vaccinated against HPV whereas 20.6% reported that they were vaccinated (106/515), the others did not provide a valid answer. HPV knowledge did not vary by age (*p* = 0.637, data not shown). Vaccination status did vary by age (*p* < 0.001), with a higher proportion of vaccinated women in younger age (18-29 years: 52.8% [56/106]).

After reading a brief explanation about CC detection and screening methods (clinician-administered test [Pap smear] or self-sampling), patients were asked if they thought that self-sampling was a good solution to reach under-screened women and nearly all agreed (93.4%, 481/515).

Women were then asked their opinion about statements comparing self-sampling and the clinician-administered test, assuming that both samples have similar accuracy. Forty-four percent (227/515) considered that a sample taken by a doctor would be more effective than a self-sample whereas 57.7% (297/515) thought that women may prefer to take a self-sample instead of going to a doctor. Three hundred and sixty-three of the 515 respondents (70.5%) expected that women may prefer urine collection as most popular self-collection method, 6.4% (33/515) may prefer vaginal self-collection, 4.9% (25/515) urine or vaginal self-sampling, 8.9% (46/515) may have no preference and the others (9.3%, 48/515) did not provide a valid answer. Opinions of women regarding self-sampling as a method to collect specimens for CC screening did not differ by age (*p* = 0.627, data not shown).

### Experiences and intentions regarding possible use of self-samples in the future

The level of understanding of the instructions on how to use the collection devices is described in Table 5. The large majority of women (> 95%) indicated that the instructions were clear for all the used self-collection devices.

**Table 5:** Clarity of instructions for self-sampling, *N =* 515 [n (%)]

No.	Question	No	Yes	No opinion	Missing value
**5**	*Did you find the instructions for urine sampling with the Colli-Pee device clear?*	16 (3.1)	491 (95.3)	1 (0.2)	7 (1.4)
**6**	*Did you find the instructions for vaginal self-sampling with the cotton swab*^a^*clear?*	3 (0.6)	502 (97.5)	3 (0.6)	7 (1.4)
**7**	*Did you find the instructions for vaginal self-sampling with the plastic brush*^b^*clear?*	11 (2.1)	494 (95.9)	2 (0.4)	8 (1.6)

^a^Vaginal Multi-Collect swab (Abbott GmbH & Co. KG, Wiesbaden, Germany)

^b^Vaginal plastic brush: Evalyn-Brush (Rovers® Medical Devices B.V., Oss, The Netherlands) or Qvintip (Aprovix AB, Uppsala, Sweden)

Nearly all women considered self-sampling with any devices as easy. (Table 6) Nevertheless, a proportion found self-sampling unpleasant (9.5% for urine, 18.6% for vaginal swab, 15.5% for vaginal plastic brushes). The large majority of women agreed that self-sampling was not painful (94.6% for urine, 88.7% for the vaginal swab, 88.7% for vaginal plastic brushes). More than 90% of women were confident that they performed the self-sampling correctly.

**Table 6:** Experience of women regarding the use of the self-sampling devices, *N =* 515 [n (%)]

**No.**	**Question**	**Fully agree**	**Partly agree**	**Don’t agree**	**No opinion**	**Missing value**
**8**	*How was your experience regarding the urine sampling with the Colli-Pee?*					
**8a**	The sampling was easy	460 (89.3)	44 (8.5)	7 (1.4)	1 (0.2)	3 (0.6)
**8b**	I found the sampling unpleasant	10 (1.9)	39 (7.6)	443 (86.0)	14 (2.7)	9 (1.8)
**8c**	The sampling was painful	5 (1)	9 (1.8)	487 (94.6)	4 (0.8)	10 (1.9)
**8d**	I think that I executed the sampling correctly	459 (89.1)	35 (6.8)	3 (0.6)	5 (1)	13 (2.5)
**8e**	I would recommend this to my friends/family	398 (77.3)	66 (12.8)	10 (1.9)	35 (6.8)	6 (1.2)
**8f**	I find urine sampling at home easier than a Pap smear, because then I don’t need to go to the doctor	275 (53.4)	150 (29.1)	42 (8.2)	44 (8.5)	4 (0.8)
**No.**	**Question**	**Fully agree**	**Partly agree**	**Don’t agree**	**No opinion**	**Missing value**
**9**	*How was your experience regarding the vaginal self-sampling with the cotton swab*^a^*?*					
**9a**	The sampling was easy	441 (85.6)	59 (11.5)	10 (1.9)	1 (0.2)	4 (0.8)
**9b**	I found the sampling unpleasant	22 (4.3)	74 (14.4)	397 (77.1)	13 (2.5)	9 (1.8)
**9c**	The sampling was painful	12 (2.3)	36 (7.0)	457 (88.7)	2 (0.4)	8 (1.6)
**9d**	I think that I executed the sampling correctly	412 (80.0)	73 (14.2)	8 (1.6)	12 (2.3)	10 (1.9)
**9e**	I would recommend this to my friends/family	359 (69.7)	92 (17.9)	27 (5.2)	31 (6.0)	6 (1.2)
**9f**	I find self-sampling with a cotton swab at home easier than a Pap smear, because then I don’t need to go to the doctor	262 (52.2)	142 (27.6)	56 (10.9)	42 (8.2)	6 (1.2)
**No.**	**Question**	**Fully agree**	**Partly agree**	**Don’t agree**	**No opinion**	**Missing value**
**10**	*How was your experience regarding the vaginal self-sampling with the plastic brush*^b^*?*					
**10a**	The sampling was easy	433 (84.1)	65 (12.6)	10 (1.9)	2 (0.4)	5 (1.0)
**10b**	I found the sampling unpleasant	18 (3.5)	62 (12.0)	413 (80.2)	11 (2.1)	11 (2.1)
**10c**	The sampling was painful	9 (1.8)	36 (7.0)	457 (88.7)	1 (0.2)	12 (2.3)
**10d**	I think that I executed the sampling correctly	402 (78.1)	75 (14.6)	10 (1.9)	14 (2.7)	14 (2.7)
**10e**	I would recommend this to my friends/family	358 (69.5)	102 (19.8)	15 (2.9)	32 (6.2)	8 (1.6)
**10f**	I find the self-sampling with a plastic brush at home easier than a Pap smear, because then I don’t need to go to the doctor	268 (52.0)	143 (27.8)	53 (10.3)	44 (8.5)	7 (1.4)

^a^Vaginal Multi-Collect swab (Abbott GmbH & Co. KG, Wiesbaden, Germany)

^b^Vaginal plastic brush: Evalyn-Brush (Rovers® Medical Devices B.V., Oss, The Netherlands) or Qvintip (Aprovix AB, Uppsala, Sweden)

Participants reported also their intention to recommend the self-sample to their friends and/or family for each device (90.1% for urine, 87.6% for vaginal swab, 89.3% for vaginal plastic brushes).

Approximately 80% of participants indicated that taking a specimen themselves was more comfortable than going to a doctor for taking a Pap smear.

All answers related to women’s experience (ease of performance, unpleasant feelings, experience of pain) were symmetrical over the diverse self-sampling procedures regarding the two vaginal plastic brushes (Evalyn-Brush or Qvintip) (*p*-values for symmetry tests varying from 0.33 to 0.98).

**Fig. 1:**
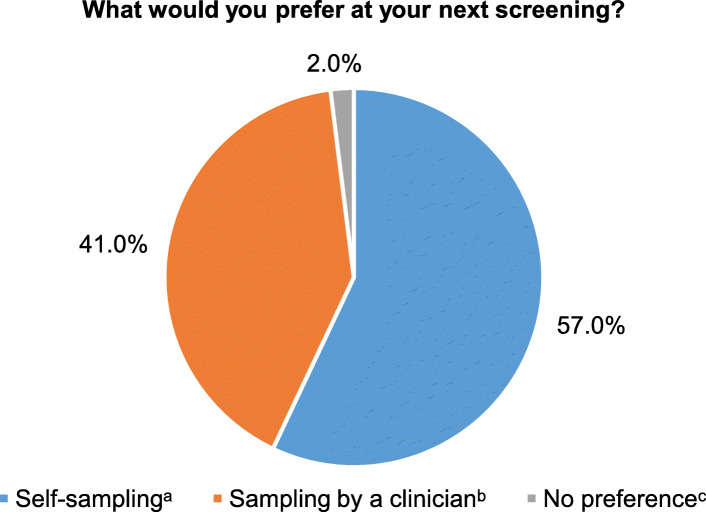
Patients’ preferences for their next screening: self-sampling or sampling by a clinician (*N* = 505). ^a^Self-sampling (*n* = 288/505, blue pie). ^b^Sampling by a clinician (*n* = 207, orange pie) [197 by gynaecologist (gyn), 8 by general practitioner (GP), 2 by either[ GP or gyn]]; ^c^No preference (*n* = 10/505, grey pie)

**Fig. 2:**
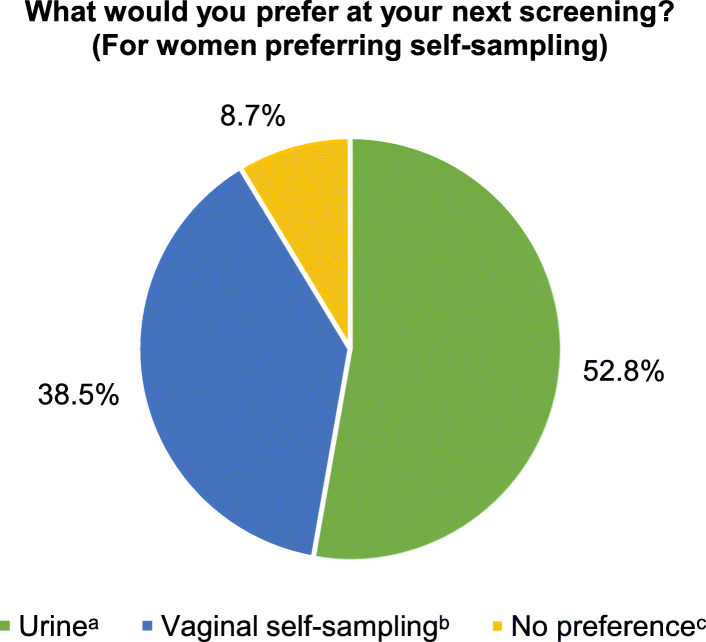
Choice of devices among women preferring self-sampling at their next screening (*N* = 288). ^a^Urine collection (*n* = 152/288, green pie); ^b^Vaginal self-sampling (*n* = 111/288, blue pie); ^c^No preference (*n* = 25/288, yellow pie)

The pie chart in Fig. 1 displays the preferences of women regarding the next screening after experiencing self-sampling. Fifty-seven percent (288/505) would opt to take a self-sample at home, 41% (207/505) would contact a gynaecologist or general practitioner (GP). Among the 288 opting for self-sampling, 152 (52.8%) would prefer collection of a urine specimen and 111 (38.5%) would prefer to take vaginal sample (Fig. 2). Relatively more women in the middle age group (30-44 years) expressed a preference for self-sampling compared to younger and older women (*p* = 0.054, data not shown). The preferences for urine or vaginal self-sampling did not vary by age (*p* = 0.478, data not shown).

## Discussion

A large majority of women completing the VALHUDES questionnaire agreed that the offer of HPV self-sampling devices may be an appropriate strategy to reach under-screened populations, regardless of the age of the patients. As patients invited for colposcopy, most patients were well aware about the role of HPV in cervical disease. Indeed 70% of participants answered they were aware of HPV infection is the cause of cervix cancer. This is a similar proportion with such knowledge as observed in a Belgian colposcopy survey after introduction of HPV vaccination in 2007 (78% vs 50% before introduction) [17, 18].

Through different statements, we have seen that the majority of women (> 95%) have a positive attitude towards self-sampling for both urine and vaginal collection. Few women found self-sampling unpleasant, difficult to perform or painful. Slightly more than half a of the participants indicated they would prefer self-sample collection at home (urine or vaginal) for their next screening over collection performed by a physician at the medical office. Nevertheless, some women showed hesitancy and would prefer collection by a physician. The reasons why women prefer collection at home instead of contacting a gynaecologist or a GP were not explored in our study. Among women preferring self-sampling, urine collection was considered more acceptable than vaginal self-collection. Previous observations by Van Keer et al. (2018), in which 124 women received a FV urine device, reported that the majority of the participants preferred FV urine collection at home (94%) over collection by a physician [19]. Our results are also in line with Leeman and colleagues (2017) exploring the acceptability and preference about specimen collection among 91 women referred for colposcopy who answered to a questionnaire using different measurement scales. When rating sampling methods on a scale from 1 to 10, the average scores were 7.6 out of 10 for clinician sampling, 8.1 for vaginal self-sampling and 8.6 for urine collection [20].

The comprehensive analysis of Belgian Individual Health Insurance Data comprising all reimbursed of Pap smears collected in 2002-2006 showed that cervical cell specimens were collected by a GP and gynaecologist, respectively in 11 and 89% of the cases [2]. The VALHUDES confirms that for 2018-2019, the gynaecologist is still the main health professional taking the specimens for cervical cancer screening in Belgium (Fig. 1).

Our findings support conclusions of previous review that offering self-sampling kits sent to under-screened women may result in increased attendance compared to traditional invitation letters where women are proposed to contact a health professional for the collection of a cervical sample. The World Health Organization (WHO) launched a global initiative, endorsed by European health authorities and stakeholders, to eliminate CC through three important steps: vaccination, screening and treatment of pre-cancer and cancer [21, 22]. According to WHO, providing women with the option of self-sampling increases acceptability and access to services. Because of its high level of performance, countries should ideally transition to HPV testing as the primary method of screening for CC [23]. Self-sampling offers an opportunity to continue safe cervical cancer screening in the context of the current COVID-19 pandemic, where lockdown measures and fear of SARS-CoV-2 contamination have limited access to health services [24].

Some limitations of our study should be acknowledged. The VALHUDES study was designed first of all to address accuracy hypotheses of HPV testing on self- versus clinician-taken samples [14]. The choice for a colposcopy setting ensured the statistical power for addressing sensitivity questions and had a minimal risk of partial verification bias inherent to screening settings. Since enrolled women are not representative for a typical screening population, the results of the questionnaire should be interpreted cautiously. Nevertheless, the VALHUDES findings are in line with a recent survey among women who actively declined participation in the regular cancer screening programme, where 70% showed interest in HPV self-sampling [3]. We must also be aware that answers might for a certain degree be biased towards plausible expectations and that communicated intentions not necessarily correspond with future behaviour. In a few cases it was seen that women answered they have read and clearly understood the instructions of the self-sampling devices, while they did not seem able to handle the device correctly (see Appendix II Supplementary Figures). Dichotomous questions were mainly used in the questionnaire to simplify the survey experience. However, this mode of questioning has the drawback of not perceiving nuances in the answers. Another weakness of the study was that, with the exception of age, no information was collected about other factors determining participation at screening. A strength of the VALHUDES study is the fact that all participating women had all self-samples taken and provided a reliable opinion about their own experiences.

Although sending self-sampling kits to the woman’s home address is more effective than traditional invitation letters to trigger participation in screening, still a substantial number of devices remains unused [11]. Substantially higher responses were notated when health professionals offer the self-sample device directly to women as observed in Latin-America [11] and in a Belgian trial in GP practices [25]. Whether these experiences might be reproducible at population scale and whether urine collection may be more effective, vaginal self-sampling should be investigated in population-based trials.

## Conclusions

We conclude that both urine and vaginal self-samples are well accepted by women, with a preference for urine sampling. Although the large majority of women are confident in their ability to perform self-sampling, four to five over ten women preferred specimen collection by a clinician.

## Supplementary Information

**Additional file 1.**

## Data Availability

Not applicable.

## Abbreviations

AGC: Atypical glandular cells
ASC-H: Atypical Squamous Cell where a High-grade lesion cannot be excluded
ASC-US: Atypical squamous cells of undetermined significance
CC: Cervical cancer
CHU de Liège: Liège University Hospital
FV: First-void
GP: General practitioner
gyn: Gynaecologist
HPV: Human papillomavirus
RZ Tienen: General Regional Hospital Heilig Hart Tienen
UZA: Antwerp University Hospital
UZ Brussels: Brussels University Hospital
UZ Ghent: Ghent University Hospital
VALHUDES: VALidation of HUman papillomavirus assays and collection DEvices for Self-samples and urine samples
WHO: World Health Organization

## References

[1] Landy R (2020). Impact of screening on cervical cancer incidence: a population-based case-control study in the United States. Int J Cancer.

[2] Arbyn M (2014). Attendance at cervical cancer screening and use of diagnostic and therapeutic procedures on the uterine cervix assessed from individual health insurance data (Belgium, 2002-2006). PLoS One.

[3] Bennett KF, et al. Barriers to cervical screening and interest in self-sampling among women who actively decline screening. J Med Screen. 2018;25(4):211-7. 10.1177/0969141318767471. 10.1177/0969141318767471 PMC6262593 29649936

[4] Ostensson E (2015). Barriers to and facilitators of compliance with clinic-based cervical cancer screening: population-based cohort study of women aged 23-60 years. PLoS One.

[5] Vorsters A (2017). Overcoming barriers in HPV vaccination and screening programs. Papillomavirus Res.

[6] Blomberg K (2008). How do women who choose not to participate in population-based cervical cancer screening reason about their decision?. Psychooncology.

[7] Arbyn M (2012). Evidence regarding human papillomavirus testing in secondary prevention of cervical cancer. Vaccine.

[8] Ronco G (2014). Efficacy of HPV-based screening for prevention of invasive cervical cancer: follow-up of four European randomised controlled trials. Lancet.

[9] Arbyn M, Castle P (2015). Offering self-sampling kits for HPV testing to reach women who do not attend in the regular cervical cancer screening program. Cancer Epidemiol Biomark Prev.

[10] Arbyn M (2014). Accuracy of human papillomavirus testing on self-collected versus clinician-collected samples: a meta-analysis. Lancet Oncol.

[11] Arbyn M (2018). Detecting cervical precancer and reaching underscreened women by using HPV testing on self samples: updated meta-analyses. BMJ.

[12] Pathak N (2014). Accuracy of urinary human papillomavirus testing for presence of cervical HPV: systematic review and meta-analysis. BMJ.

[13] Sellors JW (2000). Comparison of self-collected vaginal, vulvar and urine samples with physician-collected cervical samples for human papillomavirus testing to detect high-grade sqaumous intraepethelial lesions. CMAJ.

[14] Arbyn M (2018). VALHUDES: a protocol for VALidation of HUman papillomavirus assays and collection DEvices for HPV testing on self-samples and urine samples. J.Clin.Virol..

[15] StataCorp (2019). STATA Base Reference Manual Release 16.

[16] Schafer J (1999). Multiple imputation: a primer. Stat Methods Med Res.

[17] Donders GG (2009). Change in knowledge of women about cervix cancer, human papilloma virus (HPV) and HPV vaccination due to introduction of HPV vaccines. Eur J Obstet Gynecol Reprod Biol.

[18] Donders GG (2008). Knowledge of cervix cancer, human papilloma virus (HPV) and HPV vaccination at the moment of introduction of the vaccine in women in Belgium. Arch Gynecol Obstet.

[19] Van Keer S (2018). Human papillomavirus genotype and viral load agreement between paired first-void urine and clinician-collected cervical samples. Eur J Clin Microbiol Infect Dis.

[20] Leeman A (2017). HPV testing in first-void urine provides sensitivity for CIN2+ detection comparable to a physician-taken smear or brush-based self-sample: cross-sectional data from a triage population. BJOG.

[21] Das M (2021). WHO launches strategy to accelerate elimination of cervical cancer. Lancet Oncol.

[22] Arbyn M (2021). The European response to the WHO call to eliminate cervical cancer as a public health problem. Int J Cancer.

[23] WHO (2020). Global strategy to accelerate the elimination of cervical cancer as a public health problem.

[24] Arbyn M (2020). Tackling cervical cancer in Europe amidst the COVID-19 pandemic. Lancet Public Health.

[25] Peeters E (2020). Efficacy of strategies to increase participation in cervical cancer screening: GPs offering self-sampling kits for HPV testing versus recommendations to have a pap smear taken - a randomised controlled trial. Papillomavirus Res.

