# Supplementary material for: Cervical cancer screening using HPV tests on self-samples: attitudes and preferences of women participating in the VALHUDES study
Source: Arch Public Health. 2021 Aug 30;79:155. doi: 10.1186/s13690-021-00667-4 (PMC8403820; doi:10.1186/s13690-021-00667-4)
Supplement: Supplementary file 1 — Additional file 1. [file 13690_2021_667_MOESM1_ESM.docx]

**APPENDICES**

Supplementary material related to this article can be found, in the online version, at doi: https://doi.org/10.1016/j.jcv.2018.08.006

**Appendix I Questionnaire**

**QUESTIONNAIRE BEFORE SAMPLING**

**1. Did you know – before you accepted to participate in the VALHUDES study – that cervical cancer is caused by the human Papillomavirus (=HPV), a virus that is sexually transmitted.**

□ yes

□ no

**2. Cervical pre-cancer or cancer can be detected by a Pap smear taken by a general practitioner or gynaecologist. The cells on the Pap smear are then examined under the microscope to verify presence of abnormal cells that could evolve to cancer.**

**Instead of examining the Pap smear microscopically, it is possible to identify presence of HPV in a laboratory with a HPV test. Studies have shown that the HPV test has a better accuracy and needs to be executed less frequently than the microscopic examination. An additional advantage of the HPV test is that it can be done on a vaginal sample or a urine sample that is taken by the woman herself. This self-sample is then sent in a prepaid envelope to a lab for HPV testing.**

**In certain neighbouring countries, interesting pilot studies have been done. Women who did not have a recent Pap smear, were sent a device to take a vaginal self-sample. A woman who does not participate regularly in screening has an increased risk of developing cervical cancer. Do you find self-sampling a good solution to reach more women who do not go to a general practitioner or gynaecologist for a Pap smear?**

□ yes

□ no

□ no opinion

**3. We would like to know your opinion about the following statements (assuming that the self-sample and the clinician-taken sample have similar accuracy):**

| **3a. I find that a sample taken by a doctor is better than a self-sample** | □ yes □ no □ no opinion |
| --- | --- |
| **3b. I think that most women will choose a self-sample instead of going to a doctor** | □ yes □ no □ no opinion |
| **3c. Self-sampling is good for women who don’t have taken a Pap smear** | □ yes □ no □ no opinion |

**4. I think that most women prefer the following method of self-sampling (assuming that the self-sample and the clinician-taken sample have similar accuracy):**

□ urine sample

□ vaginal self-sample

□ no preference

□ no opinion

**QUESTIONNAIRE AFTER SAMPLING**

**5. Did you find the instructions for urine sampling with the Colli-Pee device clear?**

□ yes

□ no

□ no opinion

**6. Did you find the instructions for vaginal self-sampling with the cotton swab clear?**

□ yes

□ no

□ no opinion

**7. Did you find the instructions for vaginal self-sampling with the plastic brush clear?**

□ yes

□ no

□ no opinion

**8. How was your experience regarding the urine sampling with the Colli-Pee?**

|  | Fully agree | Partly agree | Don’t agree | No opinion |
| --- | --- | --- | --- | --- |
| **8a. The sampling was easy** | □ | □ | □ | □ |
| **8b. I found the sampling unpleasant** | □ | □ | □ | □ |
| **8c. The sampling was painful** | □ | □ | □ | □ |
| **8d. I think that I executed the sampling correctly** | □ | □ | □ | □ |
| **8e. I would recommend this to my friends/family** | □ | □ | □ | □ |
| **8f. I find urine sampling at home easier than a Pap smear, because then I don’t need to go to the doctor** | □ | □ | □ | □ |

**9. How was your experience regarding the vaginal self-sampling with the cotton swab?**

|  | Fully agree | Partly agree | Don’t agree | No opinion |
| --- | --- | --- | --- | --- |
| **9a. The sampling was easy** | □ | □ | □ | □ |
| **9b. I found the sampling unpleasant** | □ | □ | □ | □ |
| **9c. The sampling was painful** | □ | □ | □ | □ |
| **9d. I think that I executed the sampling correctly** | □ | □ | □ | □ |
| **9e. I would recommend this to my friends/family** | □ | □ | □ | □ |
| **9f. I find self-sampling with a cotton swab at home easier than a Pap smear, because then I don’t need to go to the doctor** | □ | □ | □ | □ |

**10. How was your experience regarding the vaginal self-sampling with the plastic brush?**

|  | Fully agree | Partly agree | Don’t agree | No opinion |
| --- | --- | --- | --- | --- |
| **10a. The sampling was easy** | □ | □ | □ | □ |
| **10b. I found the sampling unpleasant** | □ | □ | □ | □ |
| **10c. The sampling was painful** | □ | □ | □ | □ |
| **10d. I think that I executed the sampling correctly** | □ | □ | □ | □ |
| **10e. I would recommend this to my friends/family** | □ | □ | □ | □ |
| **10f. I find the self-sampling with a plastic brush at home easier than a Pap smear, because then I don’t need to go to the doctor** | □ | □ | □ | □ |

**11. What would you prefer at your next screening for cervical cancer? (only one choice possible)**

□ to take a urine sample myself

□ to take a vaginal sample

□ to have a sample taken by my general practitioner

□ to have a sample taken by my gynaecologist

**12. Are you vaccinated against HPV?**

□ yes

□ no

□ I don’t know

**Appendix II Supplementary Figures**

| **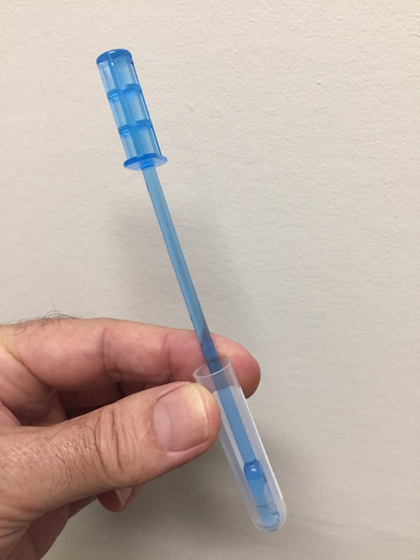** | **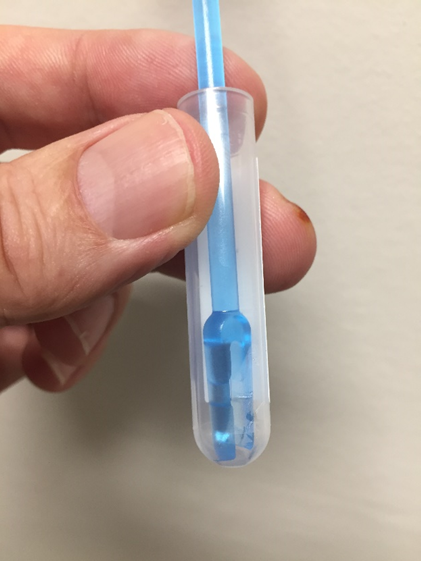** |
| --- | --- |
| **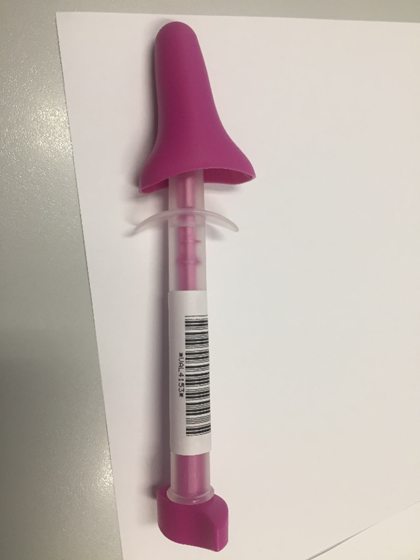** | **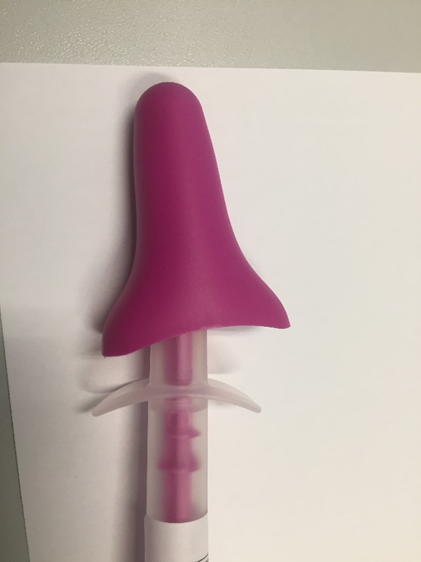** |
| **Self-sampling devices delivered by study participants after claiming they have read and understood the instruction leaflet.** In the top pictures Qvintip (Aprovix AB, Uppsala, Sweden)*,* where patient did not manage to break the tip and seal it in the tube, and on the bottom pictures Evalyn-Brush (Rovers® Medical Devices B.V., Oss, The Netherlands)*,* where patient failed to withdraw the brush inside the holder before capping. | |
